# Contralateral spontaneous rupture of the esophagus following severe emesis after non-intubated pulmonary wedge resection

**DOI:** 10.1186/s13019-020-01321-w

**Published:** 2020-10-01

**Authors:** Lei Liu, Wenbin Wu, Longbo Gong, Miao Zhang

**Affiliations:** 1 Institute of Digestive Disease, China Three Gorges University, Yichang, China; 2 Department of Gastroenterology, Yichang Central People’s Hospital, Yichang, China; 3 Department of Surgery, Xuzhou Central Hospital, 199 Jiefang South Road, Xuzhou, China

**Keywords:** Boerhaave’s syndrome (BS), Spontaneous ruptures of the esophagus, Three-dimensional CT angiography (3D-CTA), Single port, Uniportal, Video-assisted thoracoscopic surgery (VATS)

## Abstract

**Background:**

Non-intubated thoracoscopic lung surgery has been reported to be technically feasible and safe. Spontaneous rupture of the esophagus, also known as Boerhaave’s syndrome (BS), is rare after chest surgery.

**Case presentation:**

A 60-year-old female non-smoker underwent non-intubated uniportal thoracoscopic wedge resection for a pulmonary nodule. Ultrasound-guided serratus anterior plane block was utilized for postoperative analgesia. However, the patient suffered from severe emesis, chest pain and dyspnea 6 h after the surgery. Emergency chest x-ray revealed right-sided hydropneumothorax. BS was diagnosed by chest tube drainage and computed tomography. Besides antibiotics and tube feeding, a naso-leakage drainage tube was inserted into the right thorax for pleural evacuation. Finally, the esophagus was healed 40d after the conservative treatment.

**Conclusions:**

Perioperative antiemetic therapy is an indispensable item of fast-track surgery. Moreover, BS should be kept in mind when the patients complain of chest distress following emesis after thoracic surgery.

## Background

Spontaneous rupture of the esophagus, also known as Boerhaave’s syndrome (BS), typically occurs after severe emesis as a highly morbid emergency condition [1]. BS accounts for about 15% of esophageal perforations, and the tears are usually located in lower third of the esophagus [2]. Contrast esophagram and computed tomography (CT) are sufficient for the diagnosis of BS.

Non-intubated video-assisted thoracoscopic surgery (VATS) can be utilized to avoid ventilation-associated adverse effects, which has been reported to be technically feasible and safe [3]. The major complications of non-intubated procedure include intraoperative hypoxia, hypercapnia, and cough.

To our knowledge, the onset of contralateral esophageal rupture after lung resection without lymph node dissection is rare. Herein we presented a case of BS following severe emesis after non-intubated lung surgery. Meanwhile, the current evidence regarding the safety of non-intubated/tubeless thoracic surgery was reviewed briefly.

## Case presentation

**Fig. 1:**
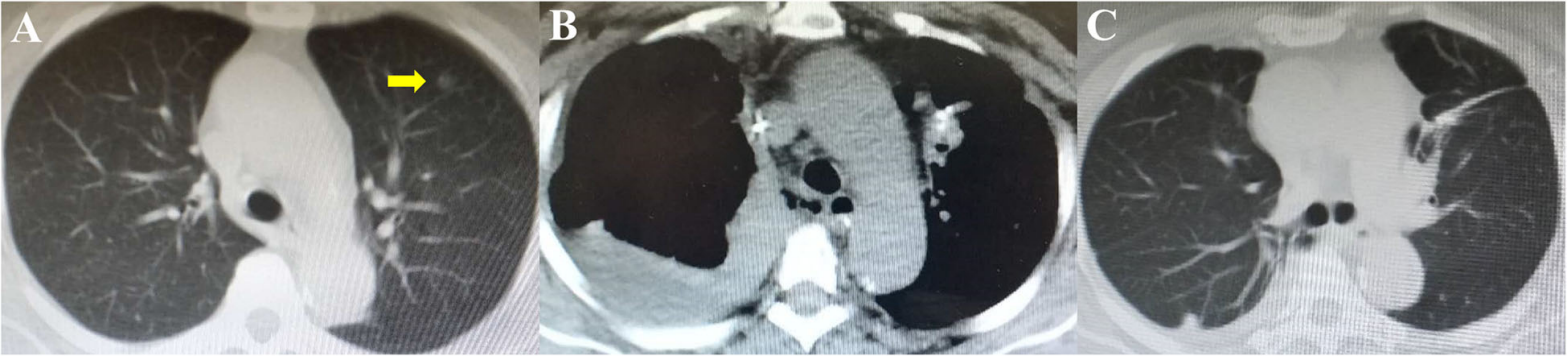
The computed tomography images. **a** A nodule in the left upper lobe was indicated by arrow. **b** The right-sided hydropneumothorax. **c** The esophagus was healed

The clinical data of the patient were treated anonymously for privacy concern. A 60-year-old previously healthy female non-smoker was admitted because the CT revealed a ground-glass nodule (GGN) about 0.5 cm in the left upper lobe (Fig. 1a). The serum neuron-specific enolase, cytokeratin-19 fragment, carcinoembryonic antigen, and squamous cell carcinoma were in normal range. After a preoperative workup, the patient was assigned to lung resection. Fast-track protocol was introduced. Preoperative three-dimensional CT angiography (3D-CTA) was established by OsiriX [4]; therefore, invasive labeling of the GGN by microcoil or hook-wire was avoided. Non-intubated uniportal VATS pulmonary wedge resection was performed under internal intercostal nerve block and targeted sedation [5, 6]. The operation time was 30 min, without obvious blood loss. Mediastinal lymph node sampling wasn’t performed because the frozen-section reported atypical adenomatous hyperplasia (AAH). Ultrasound-guided serratus anterior plane block (SAPB) using a bolus of 0.2% bupivacaine was utilized for postoperative analgesia.

Next-day discharge was scheduled because air leak was not recorded. Postoperative pathological staining of the specimen confirmed the diagnosis of AAH. The patient complained of nausea and emesis about 3 h after the operation, which was alleviated gradually after intravenous ondansetron (4 mg, once). However, 6 h after the surgery, she developed sudden tachycardia, tachypnea, dyspnea and hypotension after oral feeding. Emergency CT revealed right-sided hydropneumothorax (Fig. 1b). The turbid, yellow fluid drained from the chest tube further confirmed the diagnosis of BS.

The patient refused a timely surgical intervention. Besides antibiotics and tube feeding, endoscopy-guided naso-leakage drainage of the pleural effusion was utilized, which had been reported to be effective to rinse vomica [7]. Finally, the esophagus was healed 40d after the treatment (Fig. 1c). During the 1-year follow up, tumor recurrence or metastasis was not recorded.

## Discussion and conclusions

We identified a patient with BS after severe emesis following minimally invasive lung surgery. Severe emesis is a stressful complication of anesthesia or analgesia. A retrospective study presented 10 patients with esophageal perforation after emesis associated with large volume of food and alcohol intake [8]. Moreover, every perforation was longitudinal tears (about 1–4 cm), locating in the left lower-third of the esophagus. Then the authors hypothesized that esophageal perforation probably resulted from emesis through a pathophysiological reaction within the upper digestive tract. Furthermore, they proposed that BS should be defined as post-emetic esophageal perforation. Therefore, we concluded that the present BS was probably secondary to the uncontrolled emesis rather than the surgical procedure itself.

The incidence of post-discharge emesis after ambulatory surgery is approximately 30% [9]. Chest pain and emesis always suggest the onset of BS, but the patients don’t always present with typical clinical features. The major treatment options for BS were conservative, endoscopic and surgical approach; whereas the survival rate of the patients using these treatments was 75, 100 and 81%, respectively [10]. Surgery should be considered especially for those who are admitted within 24 h of perforation [11]. In addition, endoscopy also plays a role in the treatment of transmural defects [12], although an evidence-based recommendation is still lacking. Besides surgical and endoscopic interventions, naso-esophageal extraluminal drainage has been reported to be effective for the treatment of esophageal leaks and subsequent mediastinal abscess [13].

On the other hand, non-intubated thoracic surgery under minimal sedation with regional anaesthesia is useful to avoid nausea and emesis [14]. However, the evidence supporting non-intubated VATS as the preferred approach for lung surgery is still limited. Previous meta-analyses show that non-intubated procedures could attenuate surgery-related stress responses and decrease postoperative complications compared to intubated surgery [15, 16]. Moreover, for patients who are considered as high-risk under intubated general anesthesia due to their compromised lung function, non-intubated procedure could be considered [17]. A meta-analysis indicates that non-intubated VATS may be a better alternative to intubated surgery [18], although it requires extra vigilance to ensure the safety of the patients [19]. The disadvantages of non-intubated thoracic surgery include cough and poor maneuverability due to the movements of diaphragm and lung [20].

We searched PubMed, Web of Science, Scopus, Embase, Europe PMC, Cochrane Library and Google Scholar for randomized controlled trials (RCTs) up to June 2020 according to the PRISMA Protocol for updated evidence of nonintubated lung surgery. Key words in title or abstract include “non-intubated” or “tubeless” or “awake” and “pulmonary” or “lung” and “surgery”. Finally a total of 13 RCTs were obtained (Table 1), which covered 627 patients who underwent non-intubated or tubeless VATS. Among them, 11 (1.8%) morbidities due to gastrointestinal reactions were recorded. Based on these findings, non-intubated VATS is technically feasible and safe; however, the results should be interpreted with caution due to small samples in the trials and potential publication bias. Well-designed studies are warranted. The registered trials of non-intubated thoracic surgery were listed in Table 2, which might further elucidate the specific indications and contraindications of tubeless thoracic surgery.

**Table 1:** The reported randomized clinical trials regarding non-intubated thoracoscopic lung surgery

First author, year	Sample	Age, year	Anaesthesia method	Surgical procedure	Conversion to intubation	Postoperative analgesia	Morbidity due to gastrointestinal reactions
Pompeo, 2004 [21]	30	60 (45–68)	TEA at T4-T5	Pulmonary nodule resection	4 (13.3%)	TEA	NR
Pompeo, 2007 [22]	21	28 ± 14	Locoregional anaesthesia	Bullectomy	0	TEA	1 (4.8%)
Vanni, 2010 [23]	25	57 (51–62)	TEA	NR	0	PCIA	0
Tacconi, 2010 [24]	11	48 (43–55)	TEA	Lung nodule resection, bullectomy, pleura-lung biopsy	0	PCIA	0
Pompeo, 2011 [25]	32	64 ± 9	TEA at T4–5	Lung volume reduction	2 (6.3%)	NR	0
Pompeo, 2013 [26]	20	67 ± 12	TEA at T4	Pleurodesis	0	NR	0
Cai, 2013 [27]	30	23.5 ± 10.6	Laryngeal mask anesthesia	Bullectomy	0	PCIA	3 (10.0%)
Wang, 2014 [28]	50	43.2 ± 14.7	General anesthesia; laryngeal mask	Bullectomy, lobectomy, biopsy, mediastinal mass excision	0	NR	0
Liu, 2015 [29]	167	NR	TEA	Wedge resection, lobectomy	0	NR	4 (2.4%)
Chen, 2016 [30]	85	23.3 ± 6.8	Intravenous anesthesia	Sympathectomy	0	NR	0
Mao, 2018 [31]	30	21 ± 3.2	General anesthesia + laryngeal mask	NUSS procedure	0	PCIA	3 (10.0%)
Hwang, 2018 [32]	21	17 (17–45)	Sedation anesthesia	Bullectomy	0	Local analgesia	0
Mogahed, 2019 [33]	35	42.9 ± 9.6	General anaesthesia	Lung resections, excision/biopsy of mediastinal mass, foreign body extraction and pericardial window.	0	Intramuscular ketoprofen	NR
	35	43.5 ± 10.5	General anaesthesia + TEA				
	35	44.0 ± 9.3	General anaesthesia + intercostal block infiltration				

Abbreviations: *TEA* thoracic epidural anesthesia; *PCIA* patient controlled intravenous analgesia; *NR* not reported

**Table 2:** The registered trials of non-intubated or tubeless thoracoscopic lung surgery

Registration identifier	Year	Disease	Anaesthesia method	Estimated enrollment	Major outcomes	Status	Country
NCT00566839	2007	Emphysema	TEA	60	Mortality, FEV1, dyspnea index	Completed	Italy
NCT01469728	2011	NR	TEA	40	Grade of medical care	Completed	Italy
NCT01677442	2011	NR	TEA at the T5/T6	500	Recovery time	Unknown	China
NCT01533233	2012	Lung cancer	NR	100	Complication and morbidity	Unknown	China
NCT02109510	2014	Pneumothorax	Sedation anesthesia + intercostal nerve block	40	Postoperative discomforts	Completed	Korea
NCT02123173	2014	Lung neoplasms	NR (one lung ventilation)	71	Cardiac output	Completed	China
NCT02393664	2015	Lung neoplasms	General anesthesia + intercostal/vagal blocks	300	Quality of recovery	Unknown	China
NCT02817048	2016	Solitary lung nodule	NR (Tubeless)	100	Postoperative hospital stay	Not yet recruiting	China
NCT03275428	2017	Lung nodule	Intravenous sedation	40	Arterial oxygen pressure	Unknown	China
NCT03083080	2017	NR	Intercostal nerve plane block	30	Pain, time to lose skin sensation	Unknown	China
NCT03086213	2017	NR	Paravertebral/intercostal nerve block	48	The change of stress response markers	Unknown	China
NCT03016858	2017	Bulla	Intravenous anesthesia	320	Complications	Recruiting	China
NCT03137576	2017	Lung neoplasms	Erector spinae plane block/paravertebral block and sedation	172	Percentage of sedation escalation	Recruiting	Italy
ChiCTR-INR-17012747	2017	Thoracic diseases	General anesthesia	30	Length of hospital stay	Recruiting	China
ChiCTR-IPR-17013325	2017	Lung nodule	Intravenous anesthesia	120	CD3+, CD8+, CD4+, CD19+, NK cell concentration	Not yet recruiting	China
NCT03711461	2018	NR	NR	32	Impedance changes (swallowing)	Recruiting	China
NCT03432637	2018	Lung cancer	Spontaneous ventilating anesthesia	450	Hypoxemia or hypercapnia	Recruiting	China
NCT03471884	2018	Lung cancer	General anesthesia	82	Lung function	Recruiting	China
NCT03469323	2018	NR	NR (one-lung spontaneous breathing)	30	Quality of lung collapse	Recruiting	China
ChiCTR1800018198	2018	NR	Paravertebral nerve block + laryngeal mask	110	Glottal injury, sore throat	Recruiting	China
NCT03653494	2018	NR	General anesthesia + paravertebral block + surface spray anesthesia + vagus block with or without phrenic block	80	Anesthetic drugs needed	Enrolling by invitation	China
ChiCTR1800018204	2018	NR	Serratus anterior plane/erector spinae plane/paravertebral block	90	Nerve block time	Not yet recruiting	China
ChiCTR1800017854	2018	T1a (<2 cm) peripheral lung adenocarcinoma	NR (Tubeless)	200	Complications	Not yet recruiting	China
NCT03874403	2019	NR	Intercostal nerve block	60	The density spectral array	Recruiting	China
NCT04057586	2019	NR	NR (one lung ventilation)	240	Intraoperative cerebral oxygenation	Recruiting	China
ChiCTR1900027350	2019	Lung cancer	Intercostal/paravertebral nerve block + general anesthesia using laryngeal mask	80	Hemodynamics, general anesthetic dose, recovery time	Recruiting	China
ChiCTR1900022020	2019	Thoracic disease	General anesthesia	120	Glottal injury incidence, lung collapse score	Recruiting	China
NCT03958162	2019	Interstitial lung disease	NR (tubeless)	60	Diagnostic yield after biopsy	Not yet recruiting	China
NCT03902470	2019	Lung cancer	TEA	30	Recovery time	Not yet recruiting	Egypt

*TEA* thoracic epidural anaesthesia; *FEV1* Forced expiratory volume in one second; *NR* not reported

In summary, perioperative antiemetic with strict supervision should be considered as an indispensable item of fast-track thoracic surgery. Moreover, the occurrence of BS and a timely intervention should be kept in mind when the patients report chest distress after severe emesis following lung surgery.

## Data Availability

The data used in this report are available from the corresponding author on reasonable request.

## Abbreviations

CT: Computed tomography
BS: Boerhaave’s syndrome
VATS: Video-assisted thoracoscopic surgery
AAH: Atypical adenomatous hyperplasia
SAPB: Serratus anterior plane block
RCTs: Randomized controlled trials
